# Ubiquitin ligase A20 regulates p53 protein in human colon epithelial cells

**DOI:** 10.1186/1423-0127-20-74

**Published:** 2013-10-07

**Authors:** Jing Liu, Shaobo Yang, Zhiqiang Wang, Xiao Chen, Ziqi Zhang

**Affiliations:** 1 Department of Digestive Endoscopy, Division of South Building, Chinese PLA general hospital, Beijing 100853, China; 2 Department of Gastroenterology, Division of South Building, Chinese PLA general hospital, Beijing 100853, China

**Keywords:** Colon, Cancer, Ubiquitin E3 ligase A20, P53 protein, Colon polyp

## Abstract

**Background:**

Intestinal polyps may further develop into colon cancer; the pathogenesis is not clear. The p53 gene is an important anti-cancer gene in the body, which is suppressed in cancer. The ubiquitin E3 ligase A20 (A20) plays a role in regulating the activities of epithelial cells. This study was designed to investigate the role of the colon polyp epithelium-derived A20 in the pathogenesis of colon cancer.

**Results:**

Eighty-eight colon cancer patients and 136 colon polyp patients were recruited into this study. Human colon cancer tissue, the epithelium of adenomas polyp and hyperplastic polyp showed high levels of A20, which had a positive correlation with the cancerous tendency of colon polyps. The levels of A20 were much higher in the adenomas and hyperplastic polyps than that in the inflammatory polyps; the latter showed less cancerous tendency. A20 bound p53 to form complexes in colon cancer tissue and colon polyps. Over expression of A20 suppresses P53 protein levels in the HEK293 cells.

**Conclusions:**

A20 may play an important role in the cancerous tendency of colon polyposis.

## Background

Colon cancer is one of the leading causes of human death in the world [1,2]. The study in the pathogenesis of colon cancer advanced rapidly in recent years; still the etiology of colon cancer is unclear. The community based colon cancer screening contributes to the early diagnosis of colon cancer, which has markedly increased the therapeutic effect of colon cancer [3]. The survival rate of colon cancer is affected by the local recurrence, lymphatic metastasis and hematogenous dissemination [4]. Immune system and molecular deregulation are considered as important factors in tumor recurrence and tumor metastasis [5,6].

Colon polyps are a common disorder in human. It is estimated, after 60 years of age, more than 50% people suffers from colon polyps [7]. The phenotypes of colon polyps include hyperplastic polyps, inflammatory polyps and adenomas polyps. Certain types of colon polyps grow large and fast and become cancerous [8]. Adenomas polyps account about 50% colon polyps [9]. How the polyp epithelium differentiate into cancer tissue is still unclear.

P53 protein is a cancer suppressor protein; it is encoded by the *TP53* gene in human. P53 protein is a crucial regulator of cell cycle and apoptotic process in the cell; it functions in the cancer prevention [10]. The gene expression disorders of p53, including mutations in exon 7 [11], codon 245 [12], conserved areas [13], and the L3 structural domain [14], are associated with the pathogenesis of colon cancer. To date, the factors causing p53 suppression are still to be investigated.

Recent studies indicate the ubiquitin E3 ligase A20 (A20, in short) plays a critical role in the immune regulation as well as in associating with the pathogenesis of cancer [15]. By promoting the tolerogenicity in dendritic cells, A20 plays a role in the induction of immune tolerance, which is a crucial drawback in cancer prevention in the body. A20 and other ubiquitin E3 ligases may be involved in the suppression of p53 function [16]. In this study, we found that the adenomas and hyperplastic colon polyps had high levels of A20, which was significantly correlated with the tumorigenesis of colon polyps.

## Methods

### Reagents

The antibodies of A20, p53 were purchased from Santa Cruz (Shanghai, China). The reagents for real time RT-PCR, Western blotting, A20 over expression and immune precipitation were purchased from Invitrogen (Shanghai, China). The HEK293 cells were purchased from China Cell Line (Shanghai, China). MG132 was purchased from Sigma Aldrich (Shanghai, China). Recombinant A20 and p53 proteins were purchased from R&D Systems (Shanghai, China).

### Patients

Patients with colon cancer, non-cancer colon polyp and IBS (irritable bowel syndrome) were recruited into this study from 2005 to 2012 at our department. The diagnosis was carried out by their physicians and pathologists. After diagnosis, the colon polyps were removed by their surgeons under colonoscopy. The colon cancer tissue and polyp epithelium (dissected under an analytical microscope) were collected in the operation room. Biopsies from IBS patients were obtained under colonoscopy. The tissue was processed for the RNA and protein extraction immediately after collection; the extracts were stored at -80°C until use. The using human tissue in this study was approved by the Human Research Ethic Committee of the China PLA General Hospital. The written, informed consents were obtained from each patient.

### Follow-up

All the patients with colon polyps were required to do follow-up visits every three months after the colonoscopy surgery.

### Quantitative real time RT-PCR (qRT-PCR)

Total RNA was extracted from the collected cancer tissue and polyp epithelium using Trizol reagent according to the manufacturer’s instructions. Two micrograms of RNA were reversely transcribed in a 20 μl reaction using random primers and Transcriptor First Strand Synthesis kit. SYBR green-based qRT-PCR was performed with a Bio-Rad MiniOpticon™ Real-Time PCR Detection System. Expression of target genes was normalized to β-actin mRNA levels. The primers of A20 were: Forward: gagagcacaatggctgaaca; reverse: tccagtgtgtatcggtgcat (NCBI: NM_006290.2).

### Western blotting

Equal amounts of total protein from each sample were separated using SDS–PAGE and transferred to nitrocellulose membranes. Membranes were then blocked with 5% skim milk in TBST (Tris-buffered saline with Tween) and incubated overnight with the primary antibodies (0.5-1 μg/ml) at 4°C. Following washes with TBST, the membranes were incubated with HRP-conjugated secondary antibodies for 1 h at room temperature. The detection was carried out using an enhanced chemiluminescence Western blotting system.

### Enzyme-linked immunoassay (ELISA)

The protein extracts or an irrelevant protein (bovine serum albumin, BSA; using as negative controls), or recombinant A20 or p53 proteins, were added to micro plates at 20 μg/ml (0.1 ml/well) in duplicate; the plate was incubated overnight at 4°C. After blocking with 5% skim milk for 1 h, the first antibodies against the target proteins was added to the wells (at 10 ng/ml; incubated for 1 h), and followed by incubating with horseradish peroxidase (HRP)-conjugated secondary antibodies (5 ng/ml; incubated for 1 h)”. Washing with TBST was performed after each incubation. The formed immune complex in the plate was developed by adding 3,3',5,5'-Tetramethylbenzidine (TMB) for 20 min; the reaction was stopped by adding 25 μl 2 M H_2_SO_4_. The optical density (OD) of each well was determined by a micro plate reader (Bio Tek, Shanghai, China). The OD value of the negative controls was subtracted from the OD values of each sample well. The results were calculated against the standard curves. The sensitive limit for A20 was 2 pg/ml, and 5 pg/ml for p53 respectively.

### Immunohistochemistry

The colon tissue was obtained from 10 colon cancer patients and 10 IBS patients. The samples were processed for cryosections and stained with anti-A20 antibodies. The samples were observed with a confocal microscope. Isotype IgG was used as a negative control.

### Overexpression of A20

DNA fragments encoding A20 were generated by polymerase chain reaction (PCR) using the human source (NCBI: NM_006290.2) sense primer (5′-cttaac**ggatcc***gccacc*tgccttgaccaggacttggg-3′) and antisense primer (5′-ggtggcg**accggt**taatgttgactcttgtgaaa-3′) (ggatcc: BamHI; accggt: AgeI; gccacc: Kozak sequence). DNAs were gel purified and ligated into BamH I/Age1-digested pcDNA3.1. The A20-plasmid was designated as the pA20. HEK293 cells were transfected with pA20 or control plasmid (cpA20; containing no A20 sequence) respectively, using the Lipofectamine 2000 according to the manufacturer’s protocols. On the next day, the cells were treated with 50 μg/ml ampicillin and exposed to fresh media containing the same concentration of ampicillin every 3 days for 2-3 weeks. Individual drug-resistant clones were collected and expanded for further identification.

### Immunoprecipitation

Immunoprecipitation was performed to detect the complexes of A20/p53 using the Dynabeads® Protein G Immunoprecipitation Kit according to the manufacturer’s instruction. The precipitation antibodies were either anti-A20, or anti-p53, or isotype IgG. Proteins in the immunoprecipitations were separated by SDS-PAGE. The membrane was stained with either anti-A20 or anti-p53.

### Statistical analysis

All data were expressed as mean ± SD. The means were compared between groups with one-way analysis of variance (ANOVA) and the Student *t* test. p value <0.05 was considered significant.

## Results

### High levels of A20 and low levels of p53 in colon cancer

**Figure 1:**
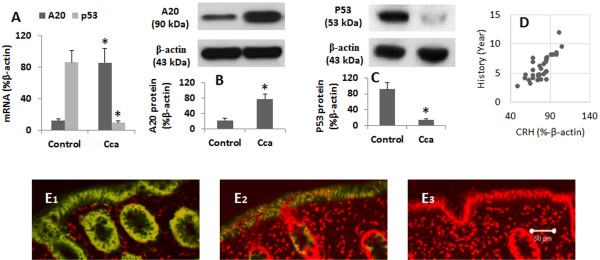
**A20 levels in colon cancer tissue.** Colon cancer tissue was collected from 88 patients with colon cancer. Control colon tissue was collected from 10 patients with IBS (irritable bowel syndrome). **A**, the bars indicate the levels of A20 or p53 mRNA (by qRT-PCR). **B** and **C**, the immune blots indicate the protein levels of A20 **(B)** and p53 **(C)**. The bars below the immune blots indicate the integrated density of the immune blots. The samples from different patients were processed individually (samples were processed in respect sample tubes in qRT-PCR and respect sample wells in Western blotting). **D**, the dot plots indicate the correlation between the polyp history and A20 levels in the polyp tissue (each dot represents one sample; some dots are overlapped). **E**, the representative confocal images indicate the A20 staining (in green; the red color indicates the nuclearing staining) in cancerous tissue (E1) and IBS colon tissue (E2). E3 is an isotype control. *, p < 0.05, compared with the control group. Samples from individual patients were processed separately.

Immune deregulation plays a role in the pathogenesis of cancer [17]; recent reports indicate that A20 contributes to immune regulation [18]. Whether A20 is involved in the pathogenesis of colon cancer is unclear. Thus, we collected 88 colon cancer tissue from the clinic (see Table 1 for the demographic data). As shown by qRT-PCR and Western blotting, the A20 levels were higher in colon cancer tissue than that in the IBS colon tissue (Figure 1A-B). The expression of p53 was also assessed in all the samples. The results showed that the expression of p53 was significantly suppressed in colon cancer tissue as compared with controls (Figure 1A, Figure 1C). When reviewed the disease history, we noted that 32/88 (36.4%) cases also suffered from colon polyp. A correlation assay was performed with the A20 levels of the colon cancer tissue and their polyp history. The results showed that the A20 levels were positively correlated with the polyp history (r = 0.766, p < 0.01. Figure 1D). By immunohistochemistry, we observed the expression of A20 was mainly localized in the epithelial cells, which was much stronger in cancerous tissue than that from the IBS colon mucosa (Figure 1E).

**Table 1:** Demographic data of pateitns with colon cancer

**Features**	**Number of patients**
Age	56.3 ± 12.6
Gender	
Male	43 (48.9%)
Female	45 (51.1%)
Histology	
Adenocarcinoma	81 (92%)
Mucinous	7 (8%)
Tumor location	
Distal	38 (43.2%)
Proximal	50 (56.8%)
Tumor grade	
Poor	35 (39.8%)
Moderate	46 (52.3%)
Well	7 (7.9%)
TNM	
T1	5 (5.7%)
T2	12 (13.6%)
T3	42 (47.7%)
T3	29 (33%)
Nodal status	
N0	48 (54.5%)
N+	40 (45.5%)
Tumor stage	
I	18 (20.4%)
II	25 (28.4%)
III	45 (51.1%)

### Levels of A20 and p53 are correlated with recurrence of colon polyp

**Figure 2:**
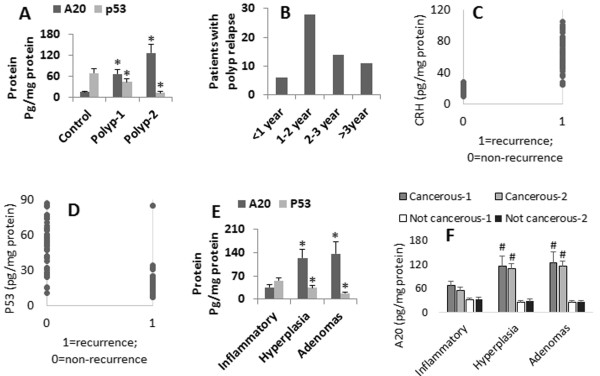
**A20 and p53 levels in colon polyps and rate of polyp relapse. A**, the bars indicate the levels of A20 and p53 in colon polyp tissue (assessed by ELISA). Polyp-1: Polyp samples were collected from 136 patients with colon polyposis. Polyp-2: Polyp samples were collected from 59 patients with relapsed polyposis. *, p < 0.05, compared with control (samples were obtained from IBS patients; see Figure 1). **B**, the bars indicate the number of patients whose polyposis relapsed. The X axis indicate the time intervals when the polyposis relapsed. **C-D**, the dot plots present the correlation between polyp history and A20 **(C)** or p53 **(D)** levels in polyp tissue with the raw data. **E**, the bars indicate the levels of A20 of p53 in colon polyps of different pathological phenotypes. **F**, the bars indicate the A20 levels in colon polyps of different pathological types of polyps. The X axis of **E** and **F** indicate the pathological types of colon polyps (the “1” indicates before the diagnosis of cancer; the “2” indicates after the diagnosis of cancer). The data in bar graphs were presented as mean ± SD. *, p < 0.05, compared with control group (IBS group; see Figure 1 in detail). #, p < 0.01, compared with adenomas group. Samples from individual patients were processed separately. Each experiment was repeated 3 times. Cancerous: The colon polyp developed into cancer.

Colon polyps have tendency to develop into colon cancer. We then recruited 136 patients with non-cancer colon polyp at the first diagnosis (the recurred cases were excluded at recruitment). The colon polyps were removed under colonoscopy. The levels of A20 in the polyp epithelium were assessed by ELISA. The results showed that the levels of A20 in the polyp epithelium were higher, p53 levels were lower, as compared to controls (Figure 2A). All the patients were followed-up three times a year. The follow-up data showed that the polyp recurred in 59/136 (43.4%) patients (Figure 2B). Their colon polyps were removed under colonoscopy again. The polyp epithelium was also examined by ELISA for the levels of A20 and p53. The results showed the levels of A20 were higher, p53 were lower, in the recurred polyp (polyp-2) than the levels in the original polyp (polyp-1) (Figure 2A). We then performed a correlation assay with the levels of A20 and p53 in the polyp and the recurrence of the original 136 patients. A significant positive correlation was identified between the levels of A20 (r = 0.87, p < 0.01. Figure 2C) or p53 (r = -0.67, p < 0.01. Figure 2D) and the recurrence of colon polyps. We further analyzed the relation between the phenotypes of the colon polyps and the levels of A20 and p53. As shown by ELISA data, the levels of A20 were higher, p53 were lower, in adenomas and hyperplastic polyps than the inflammatory polyps (Figure 2E).

### Colon polyps with high levels of A20 show tumorigenic tendency

The 136 patients with colon polyp were followed up for 3-6 years. During this period, 45 (33.1%) patients were diagnosed colon cancer. We analyzed the cancerous rate of the pathological phenotypes of the colon polyps. The results showed 4/79 (5.1%) inflammatory colon polyps, 14/21 (66.7%) hyperplastic and 27/36 (75.0%) adenomas developed into colon cancer (Table 2). The A20 levels were much higher in the cancerous group than that in non-cancerous group (IBS group) both before and after the diagnosis of cancer (Figure 2F). The data imply that the levels of A20 in colon polyps were involved in the pathogenesis of colon polyps.

**Table 2:** Demographic data of patients with colon polyp

**Features**	**Number of patients**	**Developed into cancer**
Age	**45.6 ± 14.5**	
Gender		
Male	66 (48.5%)	
Female	70 (51.5%)	
Histology		
Inflammatory	79 (58.1%)	4 (5.1%)
Hyperplastic	21 (15.4%)	14 (66.7%)*
Adenomas	36 (26.5%)	27 (75.0%)*
Location		
Distal	115 (84.6%)	
Proximal	21 (15.4%)	

*, p < 0.01, compared to inflammatory group.

### A20 binds p53 protein in colon cancer

**Figure 3:**
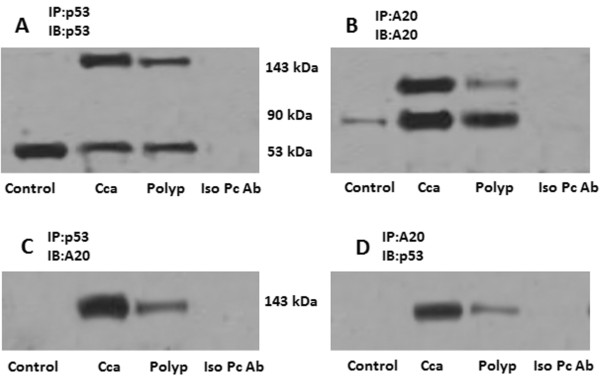
**A complex of A20 and p53.** Tissue protein was extracted from normal colon mucosa (control), colon cancer tissue (Cca) and colon polyp epithelium (polyp). The extracts were immune precipitated (IP) by anti-p53 Ab **(A, C)** or anti-A20 Ab **(B, D)**, and immune blotted (IB) with anti-p53 Ab **(A, D)** or anti-A20 Ab **(B, C)**. The immune blots indicate a complex of A20/p53 at 143 kDa. Iso pc Ab: The extracts from Cca and polyp were mixed and were precipitated by isotype IgG using as a negative control; the isotype antibody of anti-P53 was used in the experiments of panel A; the isotype antibody of anti-A20 was used in the experiments of panel B. The data represent 3 separate experiments.

The data we presented so far imply that A20 may play a role in the pathogenesis of colon cancer. The mechanism is to be further elucidated. The p53 protein is an important molecule in the prevention of tumorigenesis. Based on the above results, we wondered if A20 inhibited the p53 protein in colon cancer cells. By immune precipitation assay, we identified a complex of A20 and p53 in cancer cells as well as polyp epithelial cells with high levels of A20, but not in the polyp epithelium with low A20 levels (Figure 3).

### A20 suppresses p53 protein

**Figure 4:**
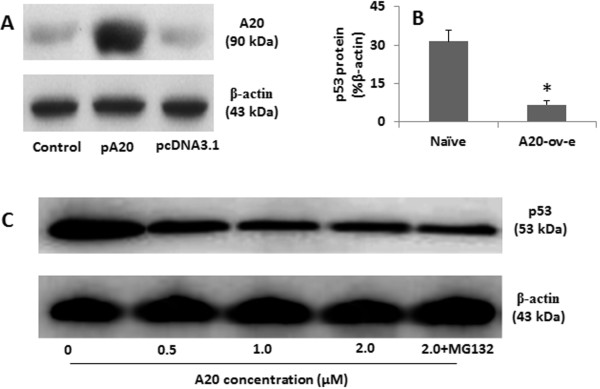
**Over expression of A20 suppresses p53 protein. A**, the immune blots indicate A20 levels in HEK293 cell extracts. Control: Naïve HEK293 cells. pA20 (or pcDNA3.1): HEK293 cells were transfected with A20 (or control) expression plasmid. The β-actin is an internal control. **B**, the bars indicate the levels of p53 in HEK293 cells (by ELISA). A20-ov-e: HEK293 cells with A20 over expression. *, p < 0.01, compared with naïve controls. **C**, the immune blots indicate the p53 levels in the cell extracts of HEK293 cells after treatment with exogenous A20 at the indicated doses (denoted below the blots) in the culture for 48 h. The data represent 3 separate experiments. The data represent 3 separate experiments.

The finding of the complex of A20 and p53 in colon cancer tissue implies that A20 may suppress p53 protein in the cells. To test the hypothesis, we over expressed A20 in HEK293 cells (Figure 4A); the expression of A20 significantly suppressed the levels of p53 in the cells (Figure 4B). To further confirm the results, we added recombinant A20 to the HEK293 cell culture. The cells were collected 48 h later. As shown by Western blotting, A20 inhibited the expression in a dose-dependent manner, which was not reversed by the proteasome inhibitor MG132 (Figure 4C).

## Discussion

The present study reports that high levels of A20 and low levels of p53 were detected in colon cancer tissue and colon polyps. The levels of A20 were significantly correlated with the cancerous tendency of colon polyps. By immune precipitation assay, we noted that A20 bound to p53 to form a complex. Over expression of A20 significantly suppressed the expression of p53 in the cells. It is well documented that colon polyps have high tendency of tumorigenesis. After removing by surgery, adenomas and hyperplastic colon polyps relapse often; some of them eventually develop into colon cancer [19-21]. Our data are in line with the previous studies [19-21] by showing that more than 70% adenomas type of colon polyps developed into colon cancer. The hyperplastic colon polyps also have a high cancerous tendency as observed in the present study. Among the recruited patients, more than 50% colon polyps are inflammatory phenotype; these colon polyps contain less A20 as compared to other two phenotypes; also the cancerous rate is much less.

Based on published data, A20 plays a role in the immune regulation. The well-documented role of A20 in the immune regulation is that A20 inhibits NF-κB activation [22]. NF-κB functions as an oncogene and the link between inflammation and cancer [23]. Other reports indicate that A20 plays an important role in the induction of immune tolerance [24,25]. It seems that A20 has multiple functions depending on the cell types and the micro environment. Recent reports indicate that intestinal epithelial cells express A20, and A20 plays a critical role in epithelial cells’ basic functions such as the antigen processing and the barrier function [26,27]. Considering that the original lesion location of colon cancer and polyp is colon epithelial cells, we inferred that A20 may play a role in these diseases. The results have confirmed our hypothesis. High levels of A20 were detected in colon cancer and colon polyp epithelium. The levels of A20 were correlated with the tumorigenesis of colon polyps.

P53 protein is a critical molecule in the maintenance of the cell homeostasis and prevention of tumorigenesis. Cumulative reports have revealed that the expression of p53 is suppressed in cancer tissue [28]. The *TP53* gene mutation is suggested as an important factor in the dysfunction of p53 that leads to tumorigenesis [11]. Our study has expanded the studies of the p53 expression by showing that the A20 binds to p53 to form complexes in colon cancer tissue and colon polyp epithelium. Such a binding leads to the suppression of p53 expression in the cells. On the other hand, MDM2 (Mouse double minute 2 homolog) is a known E3 ligase for p53. The function and regulation of MDM2 as a component of a p53-dependent negative feedback loop has formed a core paradigm in the p53 field [29]. Do MDM2 and A20 play redundant roles in human colon cancer and colon polyps is an interesting point to be further investigated.

## Conclusions

High levels of A20 in colon cancer tissue and colon polyp epithelium. Colon polyp epithelium with high A20 levels has the cancerous tendency.

## Competing interests

The authors declare that they have no competing interests.

## Author contributions

JL, SY, ZW and XC performed the experiments, analyzed experimental data and reviewed the manuscript. ZZ designed the project, supervised the experiments and wrote the paper. All authors read and approved the final manuscript.
